# Binge-drinking and household role’s associations with prevalence of domestic violence: findings from the Thailand smoking and drinking behaviour survey 2017

**DOI:** 10.1186/s13011-020-00278-2

**Published:** 2020-05-20

**Authors:** Wit Wichaidit, Sawitri Assanangkornchai

**Affiliations:** 1 Epidemiology Unit, Faculty of Medicine, Prince of Songkla University, 50 Kanchanavanich Rd., Kho Hong, Hat Yai, Songkhla Province 90110 Thailand; 2 Center for Alcohol Studies, Hat Yai, Thailand

**Keywords:** Binge-drinking, Household role, Domestic violence, Survey, Thailand

## Abstract

**Introduction:**

Alcohol consumption is associated with domestic violence, but the extent that binge-drinking and the household role of drinkers strengthens this association is unknown. We assessed the extent that binge-drinking behavior and the household role of the drinker were associated with alcohol-related domestic violence.

**Methods:**

We analyzed data from a nationally-representative census survey of 36,364 households in Thailand, of whom 17,759 households had one or more drinkers (*n* = 17,759 households). We aggregated the interview data of individuals living in the same households to create household-level attributes. We used multivariate log-binomial regression analyses to calculate prevalence ratios (PR) and measure the association between drinking behavior of household members and reported domestic violence during the previous 12 months.

**Results:**

Among households with one current drinker, households with a binge-drinker had higher prevalence of reported domestic violence than households where the drinker did not binge (Adjusted PR = 7.13; 95% CI = 4.79, 10.61), and households where the female head drank had significantly lower domestic violence compared to households where the male head drank (Adjusted PR = 0.12; 95% CI = 0.04, 0.33). Among households with two or more drinkers, households with one and two or more binge-drinkers had significantly higher prevalence of domestic violence compared to households with no binge-drinker (Adjusted PR = 2.86; 95% CI = 1.68, 4.86; and Adjusted PR = 4.62; 95% CI = 2.78, 7.67, respectively).

**Conclusions:**

Binge-drinking and household role of the drinker were associated with domestic violence at the household level. However, the study methods did not allow for disentangling of the stated associations, which limited the contribution of the study beyond its reported findings.

## Introduction

Domestic violence (also known as “family violence”) refers to violence or other abuses by one person against another in a domestic setting. Domestic violence can include physical, verbal, emotional, and sexual violence [1]. The term “family violence” refers to violence against intimate partner as well as violence against other family members, such as children and the elders. Domestic violence committed against one’s spouse and partner in an intimate relationship is called “intimate partner violence” or “IPV”. Intimate partner violence is the most common form of domestic violence, to the extent that the term “domestic violence” is often used as a synonym of “IPV” [2] and the literatures on domestic violence often focus on IPV.

Factors associated with domestic violence include geographic region and socioeconomic status [3, 4]. Socioeconomic status refers to a measure of an individual’s or a family’s economic and social position compared to others. Socioeconomic status is believed to be associated with domestic violence through availability of social support [5], lack of female empowerment and independence [6], and crisis in male identity and the need to control the woman [7]. Socioeconomic status is a difficult construct to measure, and so education and occupation are often used as proxies.

Alcohol consumption is also associated with domestic violence. Alcohol is the most commonly abused substance worldwide. Approximately 43% of the global population drink, particularly in the Americas, Europe and the Asia-Pacific regions [8]. Among current drinkers, 18.2% engaged in binge-drinking, also known as “heavy episodic drinking”, i.e., consuming 60 or more grams of pure alcohol on at least one occasion at least once per month [8]. Binge-drinking and other harmful use of alcohol resulted in approximately 3 million deaths worldwide and 132.6 million disability-adjusted life years (DALYs) in 2016, with the highest burden in low-income and lower-middle-income countries [8]. Alcohol consumption reduces inhibition, thus increasing the probability of provocation and use of violence. Most previous studies on the association between alcohol consumption and domestic violence focused on such association in the context of intimate partner violence [2, 9–14]. These studies were conducted in a wide variety of geographic areas, but each study may be limited to only women who lived in urban slums in Mumbai, India [9], only among incoming male students at a university in northwestern United States [12], only among heterosexual married and cohabiting community couples [13], or might have used data from multiple countries in sub-Saharan Africa but focused only on perpetration of IPV by men against their female partners [11]. The literatures are scarce on the association between alcohol consumption by other family members and domestic violence committed against other family members, i.e., in the context of family violence. Alcohol consumption may have a dose-response relationship with domestic violence: qualitative [15] and quantitative [16] studies suggest that binge-drinking further increases the probability of domestic violence among drinkers, but these studies were also focused on the context of IPV. It is possible that such association between binge-drinking and increased probability of domestic violence is not limited into to intimate partners, but also occurs between other household members at varying levels.

Thailand is a middle-income country in South East Asia where the majority of drinkers are men [17]. Thai drinkers reported the needs to socialize and forget about one’s financial problems as motives for drinking [18]. Drinking serves as a symbol of masculine gender identity, and alcohol is used as a tool to justify men’s IPV and violence against other family members [19]. Studies on domestic violence in Thailand in the literatures are all in the context of IPV in married or cohabitating heterosexual relationships [4, 20–22]. The prevalence of lifetime experience of IPV in Thailand varied from 16% in a survey of 1444 women in 4 provinces in 4 regions [20] to 27% in a survey of 580 women in seven Bangkok slums [4]. The prevalence of IPV in the past 12 months was 1.5% among women in a hospital-based cross-sectional study [21]. The definitions of IPV in these mentioned studies included acts of psychological, physical and sexual violence. IPV is very commonly reported among wives of drinkers with alcohol dependence (> 90%) [23], and regular alcohol use was strongly associated with IPV in urban slum communities [4]. Women who are victims of IPV also use alcohol as a tool to cope or temporality escape from domestic issues [19].

The Thailand Smoking and Drinking Behaviour Survey 2017 was a nationally-representative cross-sectional study where households were randomly sampled in various provinces, and all adult household members who gave consent to participate were interviewed with regard to drinking behaviors and problems due to drinking, including physical and verbal violence within the household. Therefore, using data from the Survey, this study assesses the extent that binge-drinking behavior and the household role of the drinker are associated with prevalence of domestic violence in households with one or more current drinkers. We hypothesize that among these households with one or more current drinkers, the number of binge-drinkers among drinkers in a household is positively associated with the probability of domestic violence within the household. We also hypothesize that among these households, the prevalence of domestic violence varies by the sex and household role of the family member who drinks. Such information can contribute to evidence-based planning of domestic violence reduction programs.

## Methods

### Study design

We used data from the Thailand Smoking and Drinking Behaviour Survey 2017, a cross-sectional survey conducted by Thailand’s National Statistical Office which included 89,184 persons age 15 years or older in 38,989 households from all 77 provinces of Thailand.

The National Statistical Office collected the survey data from 17 May - 30 July 2017 with stratified two-stage cluster sampling, using Bangkok and 12 health service regions as the first stratum, and administrative area designation (municipality and non-municipality) as the second stratum. The National Statistical Office staff then randomly selected Enumeration Areas (EAs) (the primary sampling units) from each administrative area as the first stage using probability proportional to size. The EA refers to areas designated by Thailand’s National Statistical Office (NSO) for data collectors to complete their task in the designated time. In municipality areas, an EA was a pre-defined group of 100–150 households. In rural areas, an EA was a village with pre-defined boundaries. The National Statistical Office staff then selected 20 households from each EA (the secondary sampling units) as the second stage using systematic sampling. Altogether, 2315 EAs with 46,300 households were selected to be included in the Survey [24].

In each household, the survey included data from all household members age 15 and older. The Survey interview questionnaire was divided into 5 sections: (1) Characteristics of the study area and category of the sampled households; (2) Characteristics of the household member; (3) Tobacco consumption; (4) Alcohol consumption, and; (5) Household characteristics. The section on alcohol consumption included questions on drinking behaviors and domestic problems due to alcohol within the past 12 months. Each participating household was assigned a unique household identification number (household ID). All participants from the same household shared the same household ID.

### Survey procedures

Trained data collectors from Thailand’s National Statistical Office conducted the survey. At each of the sampled household, data collectors explained to the head of household about the study and asked for the consent of the respondents and all household members age 15 and older to participate in the survey. The minimum alcohol purchasing age in Thailand, however, is 20 years. Participants acknowledged that all information provided would remain confidential and that no personal identifying information would be used or presented. The data set available to the authors was completely de-identified. Data collectors conducted face-to-face interview to each household member separately, according to established protocol, until all eligible participants who consented to participate in the survey were interviewed. The answer choices in the question regarding intra-household effects of alcohol consumption, which included verbal and physical violence, was non-specific in nature. Data collectors did not probe participants for detail of the incidence of violence when reported or make any direct intervention. There was no special procedure to corroborate the obtained information.

### Measures

#### Number of drinkers per household

We measured the number of drinkers per household using information reported by all participating household members during the survey interviews. The reported information included whether the participant had consumed alcohol within the past 12 months, and whether the participant had done any binge-drinking (taking 5 standard drinks per session) within the past 12 months. Drinking status (current vs. former or non-drinkers) was measured with Question D1: “*Have you ever drunk alcoholic beverages?*”. We considered participants who answered “*Never*” or “*Yes, but not in past 12 months*” to be never or non-current drinkers, and all others were considered to be current drinkers. Binge-drinking was measured with Question D40: “*Within the past 12 months, have you ever drunk in large quantity at one time?*”. Remarks in the Question D40 included that the large quantity refers to 5 standard drinks or more, with explanation of what 5 standard drinks entailed for each major type of alcoholic beverage. For Question D40, data collectors did not specify or define what constitutes a drinking session. We considered participants who answered “*No*” to be non-binge drinkers, and those who answered “*Yes*” and specified any frequency of such behavior to be binge-drinkers. All respondents answered questions about their drinking behavior. However, only current drinkers were asked about their binge-drinking behaviors, where every person also provided a response. Answers on binge-drinking were thus missing for those who were not current drinkers, and we considered those who were not current drinkers to be non-binge-drinkers by default, and imputed the values accordingly.

We converted the individual-level interview data of participants (i.e., whether the participant was a current drinker and whether the participant had binge-drunk within the past 12 months) into household-level attributes (i.e., number of current drinkers in the household and number of household drinkers who had binge-drunk within the past 12 months) using the *tidyr* and *dplyr* packages in R statistical software. We used household ID as the variable for aggregation of individual-level data into household-level data. We then classified the households in the study into six categories: 1) Households with no drinker; 2) One drinker – did not binge; 3) One drinker – binged; 4) Two or more drinkers – no binge-drinker; 5) Two or more drinkers – one binge-drinker; 6) Two or more drinkers – two or more binge-drinkers.

#### Sex and role of the household members who drank

In addition, we identified the sex and role of each household members who drank in each household based on the respondent’s sex and household role (in relation to the household head) as reported during the survey interview, cross-tabulated with their drinking behavior, and identified the sex and household role of the drinkers as follow: 1) male head of household; 2) female head of household; 3) male spouse of head of household; 4) female spouse of head of household; 5) male offspring of head of household; 6) female offspring of head of household; 7) other male co-habitant; 8) other female co-habitant.

#### Experience of domestic violence due to drinking among household members

We measured experience of domestic violence among household members based on each household member’s answer to question D45: “*Within the past 12 months, have you had any problem within the household due to your own or others’ consumption of alcohol?*”. Respondents were allowed to choose only one of four possible answers: “*1) None; 2) Yes, nuisance; 3) Yes, verbal violence; 4) Yes, physical violence*”; multiple answers were not allowed. Affirmative answer to the question refers to the respondent’s own victimization. The term “*nuisance*” refers to witnessing or overhearing altercations by other household members. The term “*verbal violence*” in the Thai context refers to the act of aggressively yelling or shouting, occasionally accompanied by use of profanity. The term “*physical violence*” refers to any act of assault (mild, moderate or severe). Data collectors asked the question as it appeared on the questionnaire - no other clarifications were given. Data collectors then waited for the answer from the respondent, and selected the answer choice that corresponded to the participant’s answer.

We aggregated responses regarding verbal or physical violence due to drinking in the prior 12 months from individual respondents in the same household into household-level data. We considered reporting either verbal violence or physical violence to be an act of domestic violence. We classified each household into one of the two categories: 1) Yes (i.e., at least 1 household member reported verbal or physical violence); and 2) No (i.e., no household member reported verbal or physical violence). Those who answered “*1) None*” or “*2) Yes, nuisance*” were grouped together “*1) No*”.

### Data analyses

We have two study hypotheses: 1) among households with one or more current drinkers, the number of binge-drinkers among drinkers in a household is positively associated with the probability of domestic violence within the household; 2) among households with one or more current drinkers, the prevalence of domestic violence varies by the sex and household role of the family member who drinks. To test our study hypotheses, we excluded households with no drinkers from our analyses. Of the 38,989 households that participated in the survey, 21,230 households did not have any household member who drank. Thus our analyses included 17,759 households with one or more current drinkers (*n* = 17,759 households). All 17,759 households had complete data on measures used in the analyses. We performed univariate analyses with adjustment for household-level sampling weight to present the characteristics of the study households, as well as the number of drinkers and prevalence of reported domestic violence in the household.

To assess the extent to which binge-drinking by household members was associated with domestic violence, we performed bivariate analyses using cross-tabulation. We then performed unadjusted log-binomial regression analyses. We adjusted the percentages and standard errors in all analyses for the aforementioned complex sampling strategy using the R statistical environment with *Survey* package with Taylor series linearization [25]. As geographic region, level of education and occupation are independent predictors of domestic violence [3], we also performed multivariate log-binomial regression analyses with adjustments for these potential confounders. We used the head of household’s self-reported education and occupation as the proxies for the household’s socioeconomic status. We categorized the head of household’s education into 4 groups: 1) primary education or less; 2) junior high school; 3) senior high school; 4) associate’s degree or higher. We categorized the head of household’s occupation into 5 groups: 1) agricultural workers; 2) technicians; 3) service workers; 4) executives and professionals; 5) unemployed. Multivariate analyses were stratified by number of drinkers per household (households with one drinker vs. households with two or more drinkers). To address the effect of multiple tests of statistical significance in log-binomial regression models, we used the Bonferroni method to lower the alpha value that denotes statistical significance by dividing the *p*-value of 0.05 by the number of comparisons being made in the respective model. The prevalence ratio (PR) and 95% confidence interval (CI) with the p-value below the level modified with the Bonferroni method were bolded in the table to denote statistical significance. We analyzed data using complete case analysis.

To assess the extent to which the sex and household role of the drinker were associated with domestic violence, we cross-tabulated the sex and household role of the drinker with the reported domestic violence by any household member. We stratified the analyses by number of drinkers per household (households with one drinker vs. households with two or more drinkers). In households with two or more drinkers, we assessed the difference between households where the two or more drinkers included the male head and the female spouse vs. households where the drinkers included the female head and the male spouse vs. households where the drinkers did not include both the household head and the spouse. After cross-tabulations, we performed unadjusted and adjusted log-binomial regression analyses in a similar manner as described in the previous paragraph. Among households with one drinker, households where the drinker was the male head of household were considered as the reference group. Among households with two or more drinkers, households where the drinkers included both the male head and the female spouse were considered as the reference group. Multivariate log-binomial regression analyses were similarly adjusted for geographic region, household head’s level of education, and the household head’s occupation.

This secondary data analysis has received approval from the Prince of Songkla University Faculty of Medicine Ethical Review Board on 28 February 2019, project number REC.62–054–18-1.

## Results

There were 45,296 participants living in 17,759 households with at least one drinker. When accounted for the survey design, the mean age of the participants (± standard error) was 44.3 years ±0.1 years. The majority of the participants were men (53.8% ± 0.2%), and the most common level of education was primary school or lower (46.9% ± 0.4%).

The participating households had a median of 3 members who responded to the questionnaire. Most household heads had primary education or less, and worked either in the service industry, in agriculture, or were unemployed (Table 1). Among households with one drinker, the most common sex and role of the drinker was the male household head. Among households with two or more drinkers, approximately 42% were households where the drinkers included both the head of household and their spouse. The prevalence of both verbal and physical domestic violence in the past 12 months due to drinking was very low: 3.0% of the participating households had one or more household members who reported experience of domestic violence.

**Table 1:** Characteristics of participating households, the Thailand smoking and drinking behavior survey 2017 (*n* = 17,759 households)

Characteristics	Percent ± SE, or Median (IQR)
Number of household members who responded to the questionnaire	3 (2,4)
Male head of household	69.7% ± 0.5
*Regions of Thailand*	
Bangkok	10.8% ± 0.4%
Central Region (excluding Bangkok)	28.9% ± 0.5%
North	21.1% ± 0.4%
Northeast	30.3% ± 0.4%
South	8.9% ± 0.3%
*Education of Household Head*	
Primary education (Year 6) or lower	61.4% ± 0.5%
Matthayom 3 (Year 9)	11.6% ± 0.3%
Matthayom 6 or Vocational Certificate (Year 12)	13.8% ± 0.4%
Tertiary Education (Vocational Diploma or higher)	13.3% ± 0.4%
*Occupation of Household Head*	
Agriculture	30.6% ± 0.4%
Technical trade	11.6% ± 0.4%
Service industry	32.8% ± 0.5%
Executives and professionals	6.6% ± 0.3%
Unemployed	18.4% ± 0.4%
*Number of Current Drinkers in Household*	
One	76.4% ± 0.4%
Two or more	23.6% ± 0.4%
*Self-Reported Domestic Violence due to Drinking in Past 12 Months*	
Verbal Domestic Violence (% Yes)	2.8% ± 0.2%
Physical Domestic Violence (% Yes)	0.2% ± 0.0%
Any Domestic Violence (% Yes)	3.0% ± 0.2%
*Sex and Role of the Current Drinker*	
*Among Households with One Drinker (n = 13,496 households)*	
Male household head	59.7% ± 0.6%
Female household head	8.3% ± 0.3%
Male spouse of household head	10.1% ± 0.3%
Female spouse of household head	1.5% ± 0.1%
Male child of household head	10.9% ± 0.3%
Female child of household head	2.1% ± 0.2%
Male others (extended family members and co-occupants)	6.7% ± 0.3%
Female others (extended family members and co-occupants)	0.8% ± 0.1%
*Among Households with Two or More Drinkers (n = 4263 households)*	
Drinkers included male head and female spouse	30.8% ± 0.9%
Drinkers included female head and male spouse	11.1% ± 0.6%
Drinkers included all other combinations	58.1% ± 1.0%

Binge-drinking was associated with experience of domestic violence. Among households with one drinker, households where the drinker binge-drank had significantly higher prevalence of domestic violence compared to households where the drinker did not binge-drink (4.5% vs. 0.7%, Adjusted PR = 7.13, 95% CI = 4.79, 10.61) (Table 2). Among households with two or more drinkers, households with one binge-drinker had significantly higher prevalence of domestic violence compared to households with no binge-drinkers (5.9% vs. 2.1%, Adjusted PR = 2.86, 95% CI = 1.68, 4.86). Similarly, households with two or more binge-drinkers had significantly higher prevalence of domestic violence compared to households with no binge-drinkers (9.7% vs. 2.1%, Adjusted PR = 4.62, 95% CI = 2.78, 7.67).

**Table 2:** Number of Drinkers in the Household and Experience of Domestic Violence in Past 12-Months within Household (n = 17,759 households)

Characteristics	Experience of domestic violence in past 12 months^**b**^		Crude PR (95% CI)	Adjusted PR 95% CI)^**a**^
	No	Yes		
*Households with one drinker (n = 13,496 HHs)*				
The drinker did not binge (*n* = 7888 HHs)	99.3% ± 0.1%	0.7% ± 0.1%	1.0 (Ref.)	1.0 (Ref.)
The drinker binged (*n* = 5608 HHs)	95.5% ± 0.4%	4.5% ± 0.4%	**6.72 (4.56, 9.92)**	**7.13 (4.79, 10.61)**
*Households with two or more drinkers (n = 4063 HHs)*				
Households with no binge-drinker (*n* = 1886 HHs)	97.9% ± 0.5%	2.1% ± 0.5%	1.0 (Ref.)	1.0 (Ref.)
Households with one binge-drinker (*n* = 1348 HHs)	94.1% ± 0.9%	5.9% ± 0.9%	**2.86 (1.69, 4.86)**	**2.86 (1.68, 4.86)**
Households with two or more binge-drinkers (*n* = 1029 HHs)	90.3% ± 1.2%	9.7% ± 1.2%	**4.70 (2.82, 7.83)**	**4.62 (2.78, 7.67)**

Bolded numbers indicate statistical significance after Bonferroni correction

^a^Adjusted for geographic region, household head’s occupation, and household head’s education and accounted for sampling weights in all analyses

^b^One or more persons in the household reported domestic verbal or physical violence due to alcohol consumption within the past 12 months prior to the survey

The association between household role of the drinker and domestic violence was only found among households with one drinker (Table 3). Households where the drinker was the female head of household had significantly lower prevalence of domestic violence compared to households where the drinker was the male head of household (0.3% vs. 2.6%, Adjusted PR = 0.12; 95% CI = 0.04, 0.33). No other groups had significantly different prevalence of domestic violence compared to households where the drinker was the male head of household. Among households with two or more drinkers, no statistically significant differences were found with regard to prevalence of domestic violence between households where the drinkers included the male head and female spouse, households where the drinkers included the female head and male spouse, and all other households. However, households where the drinkers included the male head of household and the female spouse had higher prevalence of domestic violence than the other groups.

**Table 3:** Association between Number of Drinkers in the Household and Experience of Domestic Violence in Past 12-Months, stratified by Role of the Drinker (n = 17,759 households)

Sex and role of the drinker	Experience of domestic violence in past 12 months^**b**^		Crude PR (95% CI)	Adjusted PR 95% CI)^**a**^
	No	Yes		
*Among households with one drinker (n = 13,496 HHs)*				
Male head of household (*n* = 7710 HHs)	97.4% ± 0.2%	2.6% ± 0.2%	1.0 (Ref.)	1.0 (Ref.)
Female head of household (*n* = 1101 HHs)	99.7% ± 0.2%	0.3% ± 0.2%	**0.12 (0.04, 0.35)**	**0.12 (0.04, 0.33)**
Male spouse (*n* = 1519 HHs)	97.4% ± 0.6%	2.6% ± 0.6%	1.02 (0.64, 1.62)	0.96 (0.60, 1.55)
Female spouse (*n* = 228 HHs)	99.2% ± 0.7%	0.8% ± 0.7%	0.33 (0.07, 1.54)	0.30 (0.06, 1.40)
Male child (*n* = 1585 HHs)	97.7% ± 0.4%	2.3% ± 0.4%	0.88 (0.58, 1.35)	0.65 (0.41, 1.05)
Female child (*n* = 252 HHs)	97.7% ± 1.5%	2.3% ± 1.5%	0.91 (0.25, 3.27)	0.63 (0.18, 2.18)
Male others (*n* = 992 HHs)	97.6% ± 0.7%	2.4% ± 0.7%	0.95 (0.52, 1.74)	0.69 (0.37, 1.26)
Female others (*n* = 109 HHs)	100.0% ± 0.0%	0.0% ± 0.0%	N/A^b^	N/A^b^
*Among households with two or more drinkers*				
Male head & female spouse (+ others) (*n* = 1267 HHs)	94.3% ± 0.9%	5.7% ± 0.9%	1.0 (Ref.)	1.0 (Ref.)
Female head & male spouse (+ others) (*n* = 291 HHs)	94.6% ± 1.4%	5.4% ± 1.4%	0.94 (0.52, 1.73)	0.92 (0.48, 1.76)
All other combinations (*n* = 2505 HHs)	95.2% ± 0.6%	4.8% ± 0.6%	0.84 (0.57, 1.22)	0.73 (0.48, 1.11)

Bolded numbers indicate statistical significance after Bonferroni correction

^a^Adjusted for region, household head’s occupation, and household head’s education and accounted for sampling weights in all analyses

^b^Excluded from the model due to perfect prediction

## Discussion

In this secondary analyses, we used a statistical package to aggregate individual-level responses from members of the same households into a household-level attributes, and assessed the extent that binge-drinking and household role of drinkers were associated with domestic violence due to alcohol consumption. Among households with one or more drinkers, we found that binge-drinking was significantly associated with domestic violence, and we found that households whereas the sole drinker was the male head of household had significantly higher prevalence of domestic violence than households whereas the sole drinker was the female head of household.

The prevalence of experience of domestic violence due to drinking of household members within the past 12 months was lower than that in a previous cross-sectional study [21]. Reported verbal domestic violence was disproportionately more prevalent than physical violence, and findings should be considered in such context. The low prevalence of domestic violence due to drinking could be partly attributed to the wording of the question itself. Participants were asked to answer only with regard to experience of domestic violence that they deemed to be attributed to drinking, and the true prevalence could have been higher had the word “due to drinking” been absent from the question. In addition, data collectors did not have the time to build rapport with the respondents, conducted the interviews in the respondent’s house, and were not instructed to prompt or probe for responses. Participants who experienced domestic violence due to drinking might not be willing or feel adequately safe to share sensitive information in such setting and context.

The data from our study indicated a possible pattern of association in the relationship between binge-drinking and domestic violence due to drinking: the more binge-drinkers among drinkers, the higher prevalence of domestic violence. The study findings expanded the findings of a previous study on the association between binge-drinking and domestic violence [16] by showing the variations by number of drinkers in the household. However, the methodology adopted in this study suggests caution in the interpretation of the findings. The majority (57.9%) of Thai binge-drinkers in the survey drank on a regular basis (at least once per week), and about one-fourth (25.7%) of binge-drinkers engaged in binge-drinking on a regular basis [17, 26]. The coding for binge-drinkers in our study did not allow us to distinguish heavy/harmful/problematic drinking. The use of validated tools such as the Alcohol Use Disorder Identification Test (AUDIT) [27] in future surveys may allow for a better measurement of drinking behaviors.

Previous studies showed that alcohol consumption can affect many individuals in the same household [28], and our study helped to make this expansion beyond the predominant context of IPV. Thai female drinkers generally drank alcohol infrequently and at low level [17], which might have contributed to the lower prevalence of domestic violence. The relatively high prevalence of violence among households where both spouses drank led to a question on whether the drinking and violence in this group were signs of maladaptive coping among spouses with history of experiencing intimate partner violence themselves [29]. Nonetheless, caution is advised against the assumption that domestic violence occurred due to alcohol use by men. In addition to drinking, factors associated with domestic violence perpetration among men include childhood trauma, witnessing domestic violence as a child, depressive symptoms, permissive attitudes for violence, and controlling behaviors [30, 31]. Furthermore, women also engage in domestic violence under various motivations [32].

A number of caveats are recommended in the interpretation of the study findings. The reported acts of domestic violence were predominantly verbal and not physical violence. We have included both physical violence and verbal violence as “domestic violence” to contextualize the findings and the study setting. However, we understand that the use of such term could be misleading, and we urge readers to interpret the findings in the context of verbal violence rather than physical violence. The question regarding the impact from drinking restricted the reported domestic violence to verbal or physical violence *due to drinking* within the past 12 months, and the data in this study did not contain details with regard to the number of incidents, the possible sequence of events between drunkenness and occurrence of domestic violence, and the nature of the violence itself. Caution is advised in the interpretation with regard to the notion of temporality or causality.

The strength of this study was the national-level representation of study households, with a sample size that conveyed adequate statistical power. However, the study had a number of limitations. Firstly, the domestic violence measurement question did not allow us to disentangle whether the drinker or binge-drinker in the household was the actual perpetrator of the domestic violence. For example, in a household with 1 binge-drinker and 1 non-binge drinker, report of domestic violence could refer to victimization from either the binge drinker or the non-binge-drinker. The findings should only be interpreted at the household level, as an association between number of self-reported drinkers and binge-drinkers in a household and report of domestic violence victimization by any member of the household. Secondly, the study questionnaire did not include details of each incident of violence, which limited our understanding of the nature of domestic violence in this study. Sexual violence was not included as an answer choice, which further limited our understanding of the association between drinking and violence. Thirdly, and most importantly, there are issues with regard to the reliability of measures. There was no standard definition of a “*drinking session*” in the binge-drinking measurement question, thus there was a possibility of misclassification. The question to measure violence was asked as it was written on the questionnaire with no attempt to probe for answers, and there was no corroboration for the obtained information, possibly leading to under-reporting and under-estimation of domestic violence. In Thailand, problems related to alcohol and domestic violence are considered shameful and often not revealed to people outside the family [19]. Studies from spouses of drinkers who have alcohol dependence and mental health problems showed much higher prevalence of domestic violence than the prevalence reported in this study [23, 33]. The question to measure domestic violence allowed for only one answer, and survey participants who experienced both verbal and physical violence might have decided to report the act as verbal violence. The National Statistical Office is an agency of the Royal Thai Government, thus the data collectors might have been perceived by some participants as being government officials, potentially contributing to under-reporting of domestic violence. The prevalence of violence in households with drinkers could have been under-estimated, and the PR could have been biased toward the null value. Lastly, the cross-sectional design of the study did not allow for temporality and it is not possible to make causal inference on the association between alcohol consumption and domestic violence.

### Recommendations for future studies

Future studies should explore the mechanisms of these associations in greater details, such as by including more detailed questions and employing qualitative research methods. Future studies should also consider measuring domestic violence in a more complete manner, and include violence that occurs without attribution to alcohol, in order to achieve a better understanding of violence within the household.

## Conclusions

We found an association between alcohol-related domestic violence victimization vs. number of binge-drinkers in the household and household role of the drinkers. However, the inability of the study methods to disentangle the relationship between binge-drinking and domestic violence limited the contribution of the study beyond its reported findings. Reported verbal domestic violence was disproportionately more prevalent than reported physical violence, and findings should be considered in such context.

## Data Availability

The datasets analyzed during the current study are available from the corresponding author on reasonable request.

## Abbreviations

IPV: Intimate partner violence
EA: Enumeration Area
NSO: Thailand’s National Statistical Office
ID: Identification number
PR: Prevalence Ratio
SE: Standard error
IQR: Inter-quartile range (1st quartile, 3rd quartile)
HH: Household
